# Psychopathy Moderates the Relationship between Orbitofrontal and Striatal Alterations and Violence: The Investigation of Individuals Accused of Homicide

**DOI:** 10.3389/fnhum.2017.00579

**Published:** 2017-12-01

**Authors:** Bess Y. H. Lam, Yaling Yang, Robert A. Schug, Chenbo Han, Jianghong Liu, Tatia M. C. Lee

**Affiliations:** ^1^Department of Rehabilitation Sciences, The Hong Kong Polytechnic University, Hung Hom, Hong Kong; ^2^Department of Pediatrics, University of Southern California, Los Angeles, CA, United States; ^3^School of Criminology and Criminal Justice, California State University, Long Beach, Long Beach, CA, United States; ^4^Nanjing Brain Hospital, Nanjing Medical University, Nanjing, China; ^5^School of Nursing, University of Pennsylvania, Philadelphia, PA, United States; ^6^Laboratory of Neuropsychology, The University of Hong Kong, Pok Fu Lam, Hong Kong; ^7^Laboratory of Cognitive Affective Neuroscience, The University of Hong Kong, Pok Fu Lam, Hong Kong; ^8^The State Key Laboratory of Brain and Cognitive Sciences, The University of Hong Kong, Pok Fu Lam, Hong Kong

**Keywords:** psychopathy, violence, orbitofrontal cortex, striatum, cortical thickness

## Abstract

Brain structural abnormalities in the orbitofrontal cortex (OFC) and striatum (caudate and putamen) have been observed in violent individuals. However, a uni-modal neuroimaging perspective has been used and prior findings have been mixed. The present study takes the multimodal structural brain imaging approaches to investigate the differential gray matter volumes (GMV) and cortical thickness (CTh) in the OFC and striatum between violent (accused of homicide) and non-violent (not accused of any violent crimes) individuals with different levels of psychopathic traits (interpersonal and unemotional qualities, factor 1 psychopathy and the expressions of antisocial disposition and impulsivity, factor 2 psychopathy). Structural Magnetic Resonance Imaging data, psychopathy and demographic information were assessed in sixty seven non-violent or violent adults. The results showed that the relationship between violence and the GMV in the right lateral OFC varied across different levels of the factor 1 psychopathy. At the subcortical level, the psychopathy level (the factor 1 psychopathy) moderated the positive relationship of violence with both left and right putamen GMV as well as left caudate GMV. Although the CTh findings were not significant, overall findings suggested that psychopathic traits moderated the relationship between violence and the brain structural morphology in the OFC and striatum. In conclusion, psychopathy takes upon a significant role in moderating violent behavior which gives insight to design and implement prevention measures targeting violent acts, thereby possibly mitigating their occurrence within the society.

## Introduction

Prior literature has shown that there is a link between brain alterations and antisocial behaviors (Raine and Yang, 2006; Yang and Raine, 2009). However, antisocial individuals itself is an inherently heterogeneous grouping which includes violent offenders, incarcerated and non-incarcerated adults with antisocial personality disorders, as well as individuals possessing different personality traits including psychopathic traits (Glenn and Yang, 2012). With reference to prior literature, offenders with various personality traits are likely to possess differential brain abnormalities (Soderstrom et al., 2002; Bertsch et al., 2013; Contreras-Rodríguez et al., 2015; Santana, 2016). For instance, Contreras-Rodríguez et al. (2015) found that psychopathic individuals had gray matter reduction involving prefrontal cortex, paralimbic, and limbic structures. On the other hand, Bertsch et al. (2013) detected alterations in orbitofrontal and ventromedial prefrontal cortex regions, and the temporal pole in antisocial individuals with borderline personality disorder while they found volumetric reductions in midline cortical areas (e.g., dorsomedial prefrontal cortex) in antisocial individuals with psychopathic traits. Therefore, by examining personality traits, specifically psychopathy in individuals who are violent, we can gain a better understanding of the future risks of antisocial behaviors (Hare, 1991).

Previous brain imaging studies (Yang and Raine, 2009) examined the OFC and striatum in psychopathic and antisocial individuals. For instance, a meta-analysis (Yang and Raine, 2009) had found reduced GMV, and activity in the right prefrontal cortex, including the OFC among antisocial individuals. Moreover, this OFC abnormality was associated with emotional deficits and poor decision-making in these individuals. Alongside with these findings, Blair (2007) also argued that the impairment in medial OFC (mOFC) might explain the dysfunction of flexible behavioral change found in psychopathy. Apart from the prefrontal cortex, the striatum, which is related to reward-seeking, is also a major brain region that is implicated in antisocial individuals (Raine and Yang, 2006). To be specific, dorsal striatum is related to habitual, or response-driven decision making while ventral striatum is generally known as a structure that is important in reward or goal-directed behaviors (O’Doherty et al., 2004; van der Meer et al., 2010). However, the striatum has received much less attention. Regarding the limited prior findings on the striatum, they were inconsistent. For instance, antisocial behaviors were associated with increased striatum GMV and activities (Tiihonen et al., 1995; Barkataki et al., 2006; Gatzke-Kopp et al., 2009; Ducharme et al., 2011; Schiffer et al., 2011). However, opposing results were found in other studies (Barros-Loscertales et al., 2006; Vollm et al., 2007). These inconsistent findings can be explained by the differences in the participants’ characteristics (e.g., age) (Gogtay et al., 2004) and the varying definitions of antisocial behavior across studies as well as the variations in personality traits (e.g., Gregory et al., 2012)., Indeed, the literature regarding the OFC also suffered from the same limitation (Blair, 2010; Glenn and Yang, 2012) and most of the studies have focused on GMV which is only one dimension of structural brain characteristics. The present study examined the specific antisocial group-homicide for two major reasons. First, it is suggested that different types of violence have different underlying psychosocial mechanisms (Acierno et al., 1999). However, prior literature investigated homicide along with other types of violence (e.g., rape) (e.g., Gregory et al., 2012). Secondly, homicide causes high financial burden to the criminal justice and mental health system (DeLisi et al., 2010). Hence, the present study studied homicide specifically instead of a variety of violent offenses (e.g., physical assault and rape). Although it is important to study this specific type of violence, prior brain imaging studies pertaining to this group of people are scarce (Raine et al., 1998; Ermer et al., 2013). Specifically, Raine et al. (1998) found that affective murderers had lower left and right prefrontal functioning, higher right hemisphere subcortical functioning, and lower right hemisphere prefrontal/subcortical ratios when compared to the controls. A more recent study (Ermer et al., 2013) suggested that psychopathic traits were related to decreased GMV in diffuse paralimbic regions such as the OFC in incarcerated male adolescents. Taken these results together, regardless of inconsistent findings and the lack of multi-modal neuroimaging methods in prior literature, the OFC and striatum have been the regions of interest (ROIs) for the investigation of antisocial behaviors. To address the literature gap, the present study compared two types of structural brain characteristics (GMV and CTh) between a control group and a homogenous antisocial group with one specific type of antisocial behavior- homicide.

Psychopathy, a 2-factor (interpersonal & unemotional features, and antisocial & impulsive features) personality construct (Hare, 1991; Frick et al., 2003), is a potential moderator of the association between violence and the abnormalities in the striatum and the OFC. There is mounting evidence showing that psychopathy is associated with structural abnormalities in both the OFC (Blair, 2003) and the striatum (Buckholtz et al., 2010; Glenn et al., 2010), as well as with antisocial behaviors. However, prior studies mainly investigated GMV lacking empirical evidences related to CTh in psychopathy. One study (Gregory et al., 2012) revealed that psychopathy moderated the GMV in the anterior rostral prefrontal cortex among violent offenders. Yet, this study did not examine neural correlates other than the GMV. It also treated the construct of psychopathy as one, singular concept (global/total psychopathy score) (Miller et al., 2011). However, since prior findings suggested that OFC GMV was associated with emotion deficits and poor decision- making (Raine and Yang, 2006; Yang and Raine, 2009) and that striatum GMV was associated with antisocial behaviors (Tiihonen et al., 1995; Barkataki et al., 2006; Barros-Loscertales et al., 2006; Vollm et al., 2007; Gatzke-Kopp et al., 2009; Ducharme et al., 2011; Schiffer et al., 2011), each sub-factor of psychopathy should be associated with different neural correlates (Soderstrom et al., 2002; Glenn et al., 2010). In fact, not only that each sub-factor is related to different neural correlates, each is related to different psychosocial correlates (Benning et al., 2003). Given that there are differential neural and psychosocial correlates for the two subtypes of psychopathy, it is essential to investigate both of them in relation to homicide, respectively, in the present study.

Despite the scarce studies, CTh, which is a structural characteristic of the brain independent from cortical surface or volume variance (Fischl and Dale, 2000), was found to be associated with violence (Narayan et al., 2007) and psychopathy (Ly et al., 2012; Wallace et al., 2014). For instance, right temporal cortical thinning was significantly related to severity of callous-unemotional traits in youths with conduct disorder (Wallace et al., 2014) while violence was related to cortical thinning in the right medial inferior frontal and right lateral sensory motor cortex (Narayan et al., 2007).

All these findings lead to the speculation that the interpersonal and unemotional features of psychopathy (factor 1 psychopathy) would a play a role in modulating the relationship between OFC GMV/CTh and violence while the antisocial and impulsive features (factor 2 psychopathy) would play a role in modulating the relationship between striatum GMV and violence. Hence, the present study aimed to go beyond prior literature by not only examining the global psychopathy (Miller et al., 2011) but also considering psychopathy as a 2-factor personality construct to investigate the relationship between psychopathy and the ROIs.

Taken together, the present study investigated the differential GMV and CTh in the OFC and striatum of violent and non-violent individuals with low and high psychopathy (global and the two psychopathic sub-factors), respectively. It was hypothesized that (1) the global psychopathy would moderate the GMV and CTh in the OFC, as well as the GMV in striatum in violent individuals; and (2) the interpersonal and unemotional features of psychopathy (factor 1 psychopathy) would moderate the GMV and CTh in the OFC, while the antisocial and impulsive features (factor 2 psychopathy) would moderate the GMV in the striatum in violent individuals.

## Materials and Methods

### Participants

Sixty seven participants (23 accused of homicide and 44 not accused of any crimes; 56 males and 11 females) aged from 19 to 68 years (mean = 34.09 years) were recruited from Nanjing Brain Hospital in Nanjing, China. Accused murderers were detainees who were undergoing forensic psychiatric evaluation and were considered as violent participants in the present study. Those who were not accused of any crimes were considered as non-violent participants. Twenty eight of the participants were diagnosed with schizophrenia. The cross-tabulation of psychopathy against violence in the present study and more details of the demographic and neural correlates of the participants were shown in **Tables 1** and **2**. University Institutional Review Board (IRB) approval was obtained at the University of Southern California and the Human Research Ethics Committee for Non-clinical Faculties of The University of Hong Kong in accordance with the Declaration of Helsinki. Approval for the study was also obtained at Nanjing Brain Hospital. Informed and written consent was obtained from all participants.

**Table 1:** Cross-tabulation of psychopathy against violence in the present study.

	Psychopathy (median split)		
	Low	High	Total
Non-violent	29	15	44
Violent	5	18	23
Total	34	33	67

**Table 2:** Demographic and neural correlates as a function of violence in the present study.

	Non-violent (*N* = 44)	Violent (*N* = 23)	Statistics	Total sample
**Demographics**				
Age (years)	32.61 (10.18)	36.91 (14.36)	*t*_65_ = -1.42, *P* = 0.16	34.09 (11.85)
Gender (% males)	84.09	82.61	χ^2^_1,__67_ = 0.024, *P* = 0.88	83.6
IQ	98.45 (16.43)	86.65 (13.39)	*t*_65_ = 2.97, *P* = 0.004^∗∗^	94.4 (16.36)
Schizophrenia diagnosis (% yes)	31.82	60.87	χ^2^_1,67_ = 5.24, *P* = 0.02^∗^	41.8
Whole brain volumes (×1000 mm^3^)	1465.78 (125.67)	1426.12 (109.13)	*t*_65_ = 1.28, *P* = 0.21	1452.164 (120.907)
Socio-economic status (SES)	52.16 (20.56)	60.48 (18.39)	*t*_65_ = -1.63, *P* = 0.11	55.01 (20.10)
Violence (%yes)	–	–	–	34.3
**Neural correlates**				
mOFC GMV (combined)^a^	90.34 (10.56)	86.38 (11.25)	*t*_65_ = 1.42, *P* = 0.16	88.98 (10.89)
Left^a^	44.17 (6.24)	42.37 (5.83)	*t*_65_ = 1.15, *P* = 0.25	43.55 (6.12)
Right^a^	46.17 (5.46)	44.02 (6.15)	*t*_65_ = 1.47, *P* = 0.15	45.43 (5.75)
lOFC GMV (combined)^a^	60.29 (8.38)	54.76 (80.16)	*t*_65_ = 2.60, *P* = 0.01^∗^	58.39 (8.62)
Left^a^	28.88 (4.06)	26.18 (4.31)	*t*_65_ = 2.53, *P* = 0.01^∗∗^	27.95 (4.31)
Right^a^	31.42 (5.42)	28.58 (4.32)	*t*_65_ = 2.17, *P* = 0.03^∗^	30.44 (5.22)
Caudate GMV (combined)^a^	50.75 (8.92)	48.24 (9.13)	*t*_65_ = 1.08, *P* = 0.28	49.89 (9.01)
Left^a^	24.64 (5.73)	24.28 (5.61)	*t*_65_ = 0.25, *P* = 0.81	24.51 (5.65)
Right^a^	26.11 (5.03)	23.97 (6.15)	*t*_65_ = 1.53, *P* = 0.13	25.38 (5.49)
Putamen GMV (combined)^a^	69.51 (16.69)	65.52 (18.13)	*t*_65_ = 0.90, *P* = 0.37	68.14 (17.16)
Left^a^	35.68 (9.81)	33.22 (8.28)	*t*_65_ = 1.03, *P* = 0.31	34.84 (9.33)
Right^a^	33.83 (8.25)	32.31 (11.81)	*t*(65) = 0.61, *P* = 0.54	33.30 (9.56)
mOFC CTh (combined)^b^	5.36 (0.42)	5.45 (0.35)	*t*_65_ = -0.84, *P* = 0.41	5.39 (0.40)
Left^b^	2.75 (0.23)	2.72 (0.20)	*t*_65_ = 0.63, *P* = 0.53	2.74 (0.22)
Right^b^	2.61 (0.24)	2.73 (0.20)	*t*_65_ = -2.04, *P* = 0.05^∗^	2.65 (0.24)
lOFC CTh (combined)^b^	5.39 (0.43)	5.43 (0.25)	*t*_65_ = -0.41, *P* = 0.68	5.40 (0.38)
Left^b^	2.71 (0.21)	2.72 (0.15)	*t*_65_ = -0.23, *P* = 0.82	2.72 (0.19)
Right^b^	2.68 (0.25)	2.71 (0.16)	*t*_65_ = -0.49, *P* = 0.63	2.69 (0.23)

*Numbers in brackets represent the standard deviation (SD); lateral orbitofrontal cortex- lOFC; medial orbitofrontal cortex- mOFC; cortical thickness- CTh; gray matter volumes- GMV. ^a^(×100 mm^3^); ^b^(×1 mm); ^∗^*P* < = 0.05, ^∗∗^*P* < = 0.01, ^∗∗∗^*P* < = 0.001*.

### Measures

#### Clinical and Major Assessments

Each participant were assessed regarding the lifetime presence of Axis I and Axis II psychopathology using the Chinese Classification of Mental Disorders Version 3 (Chinese Society of Psychiatry, Chinese Medical Association, 2001) and the Diagnostic and Statistical Manual of Mental Disorders (DSM-IV) (American Psychiatric Association, 1994) by a psychiatrist at Nanjing Brain Hospital. The results collected from all participants showed negative regarding both lifetime and current abuse/dependence of substance. Given part of the participants were diagnosed with schizophrenia which may confound with current findings, schizophrenia diagnosis would be controlled for in the analyses of the present study. Moreover, only one participant reported using anti-anxiety medications or anti-depressants.

**FIGURE 1:**
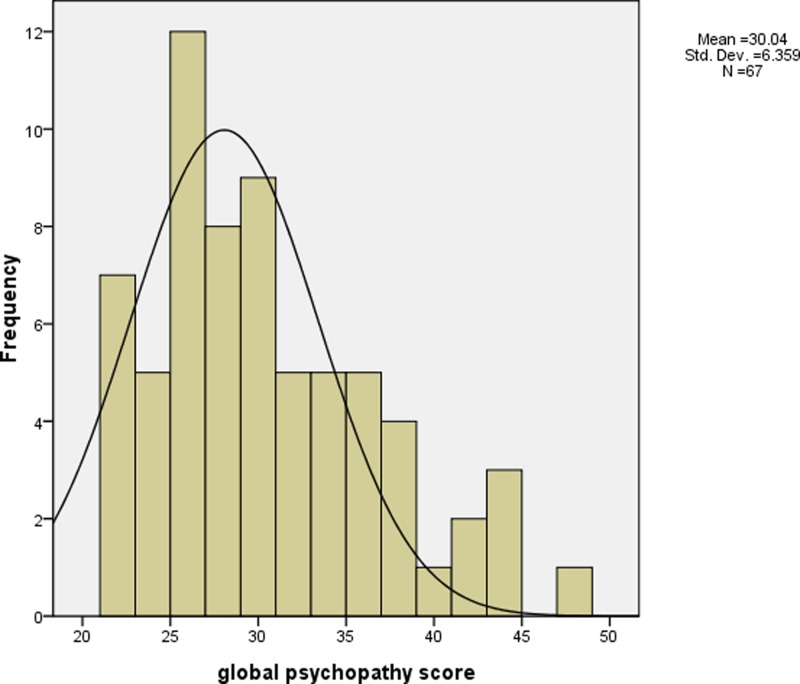
The histogram of global psychopathy *t* score (mean = 30.04 (equivalent to 4 as in global psychopathy raw score), standard deviation = 6.36).

**FIGURE 2:**
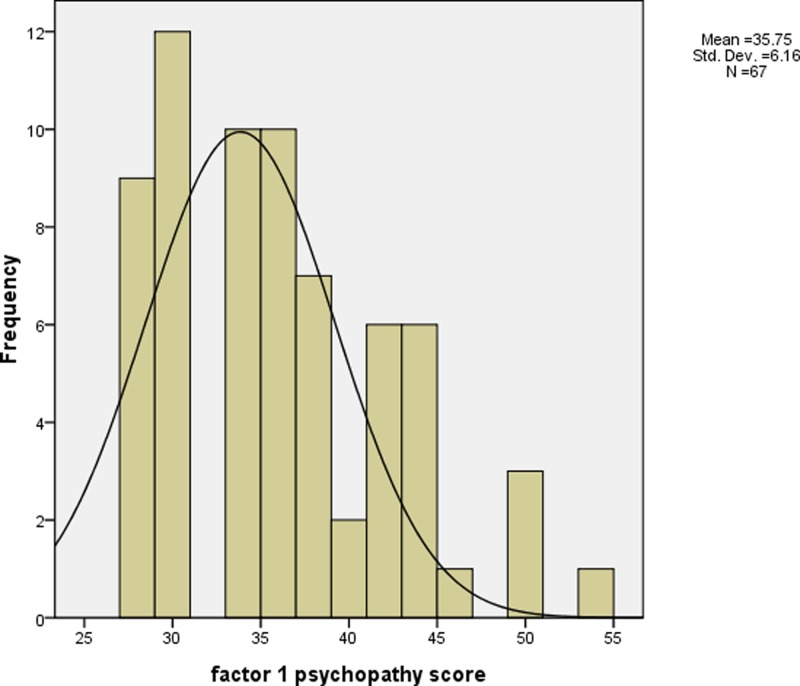
The histogram of factor 1 psychopathy *t* score (mean = 35.75 (equivalent to 3 as in factor 1 psychopathy raw score), standard deviation = 6.16).

**FIGURE 3:**
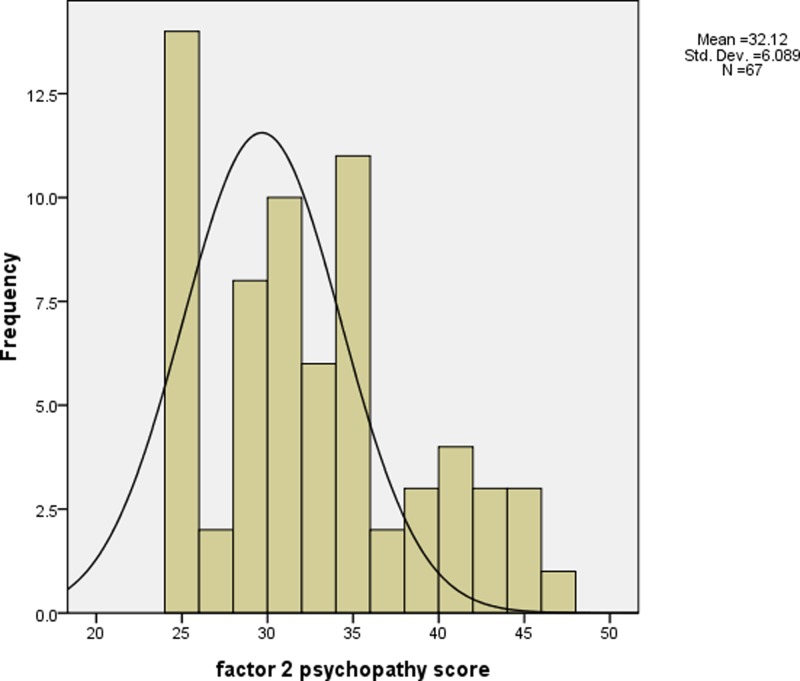
The histogram of factor 2 psychopathy *t* score (mean = 32.12 (equivalent to 2 as in factor 2 psychopathy raw score), standard deviation = 6.09).

Psychopathic traits were assessed by PCL-R (Hare, 2003). PCL-R consists of two components: factor 1, which measures personality traits, such as superficial charm, shallow affect and lack of empathy; and factor 2, which measures impulsivity and antisocial behaviors. This 2-factor structure has been well validated across different populations such as female and male criminal offenders as well as male forensic psychiatric patients (Bolt et al., 2004). Also, PCL-R obtained good interrater reliability and internal consistency previously (Vitale et al., 2002) and in the present study (*r* = 0.84). The total/ global psychopathy (the summation of factor 1 and factor 2 scores) and the two PCL-R *t* subscores (factor 1 and factor 2) were computed for analyses in the present study. **Figures 1**–**3** show the distribution of the global psychopathy *t* score, factor 1 psychopathy *t* score and factor 2 psychopathy *t* score, respectively. The expression of psychopathic traits varies across cultures (Neumann et al., 2012) which may explain why the PCL-R scores were much lower in the current sample when compared to the standard PCL-R cutoff. The full scale of IQ was measured using the Wechsler Adults Intelligence Scale: Revised in China (WAIS-RC) (Gong, 1992). SES was measured according to the scale devised by Hollingshead (Hollingshead, 1975).

#### MRI Image Acquisition and Processing

All sMRI data was acquired using a 1.5T GE Signa scanner with a single-shot gradient echo MPRAGE sequence (*TR* = 25 ms, *TE* = 6 ms, field of view = 24 cm × 24 cm, matrix = 256 × 256, flip angle = 45^°^, thickness = 1.2 mm, 124 continuous sagittal slices without gap). CTh and volumetric segmentation were estimated using the FreeSurfer software (FreeSurfer 4.0.5^1^) (Dale et al., 1999; Fischl et al., 1999, 2002), which is a widely documented and automated program for the analysis of brain structure (Fischl and Dale, 2000). Moreover, its validation was previously reaffirmed in studies regarding schizophrenia in contrast with manual measurements (Kuperberg et al., 2003).

In short, the FreeSurfer processing streams applied in the present study included three major functions: the removal of non-brain tissue, the segmentation of gray-white matter, and the transformation to a common space (i.e., the Montreal Neurological Institute space), respectively. In terms of volumetric estimation, after the FreeSurfer processing and estimation, GMV of subcortical brain ROIs in the present study including caudate and putamen were further traced manually using BrainSuite (Shattuck and Leahy, 2002), which would correct small errors. Regarding the estimation of CTh, after normalization of the intensity, gray-white tissue segmentation was used as the starting point for a deformable surface algorithm for the extraction of the pial and gray-white cortical surfaces (Dale et al., 1999). Then, the entire cortex of each participant was visually inspected for accuracies. Manual correction, if necessary, would be carried out using procedures that were established previously (Roussotte et al., 2011). After spatial normalization of the data, converting it to a cortical surface-based atlas, local CTh was estimated by calculating the shortest distance between a given point on the estimated pial surface and the gray/white matter boundary across the cortical mantle (Fischl and Dale, 2000). Maps were smoothed across the surface and averaged across participants with a 10-mm full-width at half-maximum Gaussian kernel using a non-rigid high-dimensional spherical averaging method. General linear model (GLM) was performed on the effects of each variable on thickness at each vertex for the statistical comparisons of surface maps (Fischl et al., 1999; Fischl and Dale, 2000).

### Statistical Analysis

The major aim of the present study was to investigate the moderation effect of psychopathic traits in the relationship between structural brain abnormalities (striatum and OFC) and violence. As such, the relationship between these structural brain abnormalities and violence was investigated as a function of psychopathic traits. To be specific, “structural brain abnormalities” refer to the relative differences in terms of the neural correlates between the non-violent group and the violent ones in the present study. By testing the moderation effect, we tested whether the effect of an independent variable (neural correlates) on a dependent variable (violence) varied across the level of a third variable/moderator variable (psychopathy), which interacted with the independent variable (Baron and Kenny, 1986; Edwards and Lambert, 2007). Specifically, we investigated whether the psychopathic personality traits influenced the strength of the relationship between neural correlates and violence. Therefore, the neural correlates were the independent variables and the binary variable (violence vs non-violence) was the outcome variable while the moderator was psychopathy in the present study. The ROIs (striatum and OFC) were chosen based on prior literature as well as the Qdec whole-brain findings in the present study. Based on the GMV/CTh in prior literature and the whole-brain findings devised by the vertex-based analysis using Qdec (a module of FreeSurfer developed to design and execute surface analysis), ROI analyses were performed for the lateral and mOFC (lOFC and mOFC), putamen, and caudate in the present study. The neural correlates of each ROI were computed: (1) total GMV/CTh by the summation of the left and the right hemispheric volumes/CTh; and (2) GMV/CTh by hemisphere. Correlations, between-group *t*-test and chi-square test were used to assess the association of demographic information with violence, psychopathy and neural correlates (**Tables 2**, **3**). Analyses of the ROI neural correlates were performed using SPSS (Chicago, IL, United States) via employing logistic regressions. Logistic regressions were conducted for the outcome variable (violence), with the psychopathy scores and ROI neural correlates (lOFC, mOFC, caudate and putamen), and the interaction term of the two as the predictors. Significance was set based on a two-tailed alpha level of 0.05 for all tests. IQ and schizophrenia diagnosis were significantly associated with violence in the present study (*P* < 0.05) (**Table 2**) which is consistent with prior findings (Fazel et al., 2009; Diamond et al., 2012). In all analyses, the covariates (age, IQ, sex, SES, schizophrenia diagnosis and whole brain volumes) were controlled for.

**Table 3:** Correlations and *t*-values between demographics and total neural correlates.

	mOFC GMV	lOFC GMV	Caudate GMV	Putamen GMV	mOFC CTh	lOFC CTh
Age (years)	-0.29^∗^	-0.44^∗∗∗^	-0.02	-0.30^∗^	-0.37^∗∗^	-0.32^∗∗^
Whole brain volumes (×1000mm^3^)	0.61^∗∗∗^	0.62^∗∗∗^	0.29^∗^	0.40^∗∗∗^	0.07	-0.06
Socio- economic status (SES)	-0.07	-0.08	-0.05	-0.07	0.05	-0.08
IQ	0.14	0.16	-0.12	0.08	0.13	0.16
Gender	*t*(65) = 2.68^∗∗^	*t*(65) = 3.08^∗∗^	*t*(65) = 0.62	*t*(65) = 1.96	*t*(65) = 0.43	*t*(65) = -0.71
Schizophrenia diagnosis	*t*(65) = 1.40	*t*(65) = 1.85	*t*(65) = -3.12^∗∗^	*t*(65) = 0.70	*t*(65) = 1.47	*t*(65) = 1.95
Psychopathy global score	-0.11	-0.25^∗^	0.26^∗^	0.07	-0.07	-0.11
Factor 1 psychopathy score	-0.14	-0.26^∗^	0.19	0.07	-0.10	-0.06
Factor 2 psychopathy score	-0.07	-0.23	0.29^∗^	0.05	-0.03	-0.14

*Lateral orbitofrontal cortex- lOFC, medial orbitofrontal cortex- mOFC, cortical thickness- CTh, gray matter volumes- GMV. ^∗^*P* < = 0.05, ^∗∗^*P* < = 0.01, ^∗∗∗^*P* < = 0.001*.

## Results

### Psychopathy Global Score

#### Cortical Thickness

With the CTh of the lateral and mOFC (lOFC and mOFC) for both the left and the right hemispheres, psychopathy global score and the interaction terms (e.g., lOFC × psychopathy global score) as the dependent variables, the logistic regressions showed that all CTh neural correlates (*P* > 0.05), the psychopathy global score (*P* = 0.22) and all interaction terms (*P* > 0.05) were not significant. Similar results were found without controlling for the covariates of the present study (*P* > 0.05).

#### Gray Matter Volumes

With the GMV of lOFC and mOFC, caudate and putamen in both the left and the right hemispheres, psychopathy global score and the interaction terms (e.g., caudate GMV × psychopathy global score) as the predictors, the logistic regressions showed that all GMV neural correlates (*P* > 0.05), the psychopathy global score (*P* = 0.22) and all interaction terms (*P* > 0.05) were not significant.

### Psychopathy Subscores

#### Cortical Thickness

With the CTh of the lateral and mOFC (lOFC and mOFC) for both the left and the right hemispheres, factor 1 psychopathy score and the interaction terms (e.g., lOFC × factor 1 psychopathy score) as the dependent variables, the logistic regressions showed that all CTh neural correlates (*P* > 0.05), the factor 1 psychopathy score (*P* = 0.85) and all interaction terms (*P* > 0.05) were not significant. Similar results were found for the factor 2 psychopathy model (*P* > 0.05).

#### Gray Matter Volumes

In terms of the GMV, significant main effects of neural correlates and interaction effects of neural correlates × factor 1 psychopathy (PCL-F1) were found significant in predicting violence. Specifically, the GMV neural correlates (left caudate, left and right putamen; *P* < = 0.05) and interaction terms (left caudate × PCL-F1, left putamen × PCL-F1, right putamen × PCL-F1, and right lOFC × PCL-F1; *P* < = 0.05) were significant in predicting violence. **Table 4** shows the results of logistic regressions with covariates, neural correlates (GMV), factor 1 psychopathy score and the interaction terms predicting violence. These main and interaction effects were not significant for the factor 2 psychopathy model (*P* > 0.05).

**Table 4:** Logistic regressions with covariates (step 1), neural correlates (GMV), psychopathy scores (PCL-F1), and the interaction terms (step 2) predicting violence.

	Factor 1 psychopathy score (PCL-F1) as the moderator			
	*B*	Standard error	Wald test statistics	*P*
**Step 1**				
Gender	0.97	0.94	1.06	0.30
Schizophrenia diagnosis	1.20	0.63	3.65	0.06
Age	0.001	0.03	0.003	0.96
SES	0.02	0.02	1.37	0.24
IQ	-0.04	0.02	3.49	0.06
Whole- brain volume	0.00	0.00	1.62	0.20
**Step 2**				
Gender	2.09	1.74	1.45	0.23
Schizophrenia diagnosis	4.35	2.51	3.02	0.08
Age	0.11	0.08	2.15	0.14
SES	0.10	0.06	2.61	0.11
IQ	-0.06	0.07	0.86	0.35
Whole- brain volume	0.00	0.00	3.71	0.05^∗^
Left caudate	0.04	0.02	3.80	0.05^∗^
Right caudate	0.01	0.01	0.66	0.42
Right putamen	-0.06	0.03	4.76	0.03^∗^
Left putamen	0.05	0.02	5.02	0.03^∗^
Left mOFC	0.04	0.02	2.87	0.09
Right mOFC	0.05	0.03	2.93	0.09
Left lOFC	-0.09	0.05	3.64	0.06
Right lOFC	-0.05	0.02	3.52	0.06
PCL- F1/F2	3.54	2.02	3.08	0.08
Left caudate × PCL-F1	-0.001	0.001	4.05	0.04^∗^
Right caudate × PCL- F1	0.00	0.00	0.76	0.38
Right putamen × PCL- F1	0.00	0.00	4.82	0.03^∗^
Left putamen × PCL- F1	-0.001	0.001	5.01	0.03^∗^
Left mOFC × PCL- F1	0.00	0.001	2.50	0.11
Right mOFC × PCL- F1	-0.001	0.001	2.86	0.09
Left lOFC × PCL- F1	0.002	0.001	3.68	0.06
Right lOFC × PCL- F1	0.001	0.001	3.73	0.05^∗^

*Lateral orbitofrontal cortex- lOFC, medial orbitofrontal cortex- mOFC, gray matter volumes- GMV. ^∗^*P* < = 0.05, ^∗∗^*P* < = 0.01, ^∗∗∗^*P* < = 0.001*.

## Discussion

The present study undertook a multimodal perspective in the course of investigating the role of psychopathy within the relationship of violence and the OFC and striatum (caudate and putamen). In particular, the moderation effects of the global and sub-factors of psychopathy in the context of such association were investigated with reference to two groups of participants: violent and non-violent individuals. Major findings were only consistent with the second priori hypotheses, which suggested that psychopathic traits (particularly the factor 1 psychopathy) moderated the relationship between violence and the brain structural morphology in the OFC and the striatum. Moreover, the differential results between the GMV and CTh of the OFC suggested that the neural phenotype is more sensitive at the GMV level regarding the role of psychopathy in violence. These findings help us better understand the relationship between brain abnormalities (particularly the OFC and striatum), psychopathy and violence, thus reduce the perpetration of violent crimes in the society potentially.

### Psychopathy as a Moderator

The key findings revealed that psychopathy was a moderator in the association of violence with the GMV in lateral OFC (lOFC) and striatum, its effects are particularly potent in the right hemisphere. This finding was consistent with prior findings suggesting that different personality traits played a significant role in structural brain abnormalities present within violent individuals (Soderstrom et al., 2002; Tiihonen et al., 2008; De Brito et al., 2009; Gregory et al., 2012; Bertsch et al., 2013). More specifically, the factor 1 psychopathy measuring the superficial charm, the shallow affect and the lack of empathy had moderated the association between the right lOFC GMV and violence significantly. These findings suggested that the effect of structural brain abnormality in the right lOFC on the perpetration of violent acts varied across the unemotional aspects of psychopathy. As such, the decrease of the level of unemotional aspects of psychopathy weakened the relationship between GMV in the right lOFC and the risk for the perpetration of violent acts. These findings are consistent with prior findings (Tiihonen et al., 2008; De Brito et al., 2009). Specifically, Tiihonen et al. (2008) found focal, symmetrical, bilateral areas of atrophy in GMV of the post-central gyri, frontopolar cortex, and OFC among the offenders when compared with the healthy male counterparts. Along the same line, De Brito et al. (2009) found increased GMV in the OFC and anterior cingulate cortices, as well as increased GMV in the temporal lobes bilaterally among the boys with callous- unemotional conduct problems when compared with healthy boys. It was suggested that the abnormality in OFC GMV in psychopathy and violence was due to the delay in cortical maturation in these brain areas which are involved in decision making, morality and empathy (De Brito et al., 2009). However, there was no significant finding observed for the mOFC. This might be due to the fact that lOFC and mOFC serve different functions (Iversen and Mishkin, 1970). In terms of neuroanatomical functions, OFC as a whole is involved in monitoring reward values. Specifically, lOFC is responsible for the suppression of a response that is previously associated with reward as well as the mediation of the fight-flight response to threat (Elliott et al., 2000; Blair, 2001). The significant association between the lOFC GMV and violence found in the present study suggests the dysfunctions in lOFC lead to the inability to suppress the perpetration of violence. On the other hand, mOFC is related to instrumental learning (LaPierre et al., 1995), moral reasoning (Koenigs et al., 2007) and flexible behavioral change (Blair, 2007). The present study found no significant relationship between mOFC GMV and violence which may be because the perpetration of violence is not involved in the learning process subserved by mOFC.

As for the striatum, the results showed that the association of striatum (left and right putamen and left caudate) GMV with violence varied across psychopathy, which had already been anticipated (Glenn et al., 2010). To be specific, the association between the left caudate, left and right putamen GMV and violence varied across the level of psychopathy, especially the factor 1 psychopathy (unemotional aspects of psychopathy). This finding suggested that the level of unemotional aspects of psychopathy moderated the association between the right putamen GMV and the likelihood of perpetration of violent acts. This supported prior findings, in which it was the concurred stance that psychopathy was the moderator in the relationship between brain abnormalities and violence (Soderstrom et al., 2002; Bertsch et al., 2013). However, the present finding regarding the striatum was inconsistent with a prior finding by Gregory et al. (2012). This might be because in the case of the precedent (e.g., Gregory et al., 2012), violent participants having committed a variety of violent offenses, including murder, rape and grievous bodily harm were examined; whereas the current study indexed violence by a homogenous group wherein participants who were accused only of homicide. Various types of violence have different underlying psychosocial mechanisms (Acierno et al., 1999) and these mechanisms may be reflecting different corresponding neural correlates. This may be the reason why no significant finding was found for striatum by Gregory et al. (2012) when a variety of violent acts were analyzed altogether while the present study found significant results in studying homicide alone. Taken all of the above findings together, the significant moderation effect of psychopathy on the relationship of structural abnormalities in the lOFC and striatum with violence suggested that reducing psychopathy could potentially protect against the perpetration of violent crimes. Future studies could perhaps look into this hypothesis, as it would likely facilitate the design of policies and measures of intervention and prevention in order to reduce the occurrence of violent activities in the society.

Nevertheless, how the cortical neural correlates (GMV/CTh of the OFC) related to violence did not vary according to the antisocial and impulsive psychopathic traits (factor 2). In addition to that, the antisocial and impulsive psychopathic trait (factor 2) was not a moderator in the relationship between the striatal neural correlates (GMV of the putamen/caudate) and violence. In short, these findings suggest that each sub-factor of psychopathy plays a specific role in the relationship of brain abnormalities and violence.

### Lateralization of the ROIs

It was observed that psychopathic traits moderated the OFC GMV in the right, but not the left hemisphere in the present study. This suggested that psychopathy might play a significant effect on the structure of the brain, particularly in the OFC GMV within violent individuals in the right hemisphere. This might be because antisocial behavior is related to the right-sided prefrontal pathology. Specifically, Tranel et al. (2002) opined that impairment in social conduct, decision-making, emotional processing and personality is only present in people with lesions to their right, but not the left OFC. However, the underlying mechanism of such a right-sided pathology in relation to antisocial behavior and psychopathy is yet to be investigated in future studies.

### Cortical Thickness and Gray Matter Volumes

The differential findings between the GMV and CTh of the OFC suggest that the role of psychopathy in violence might be more significant at the level of GMV when compared to the CTh. In fact, CTh is a structural characteristic of the brain, which is independent from cortical surface or volume variance (Fischl and Dale, 2000). More specifically, it is derived from a surface modeling (unfolding and flattening) (Fischl and Dale, 2000). There are a number of advantages in using this technique in comparison with the GMV measure. For instance, the measure of CTh was deemed more sensitive in neurodegenerative diseases such as in Parkinson’s disease (Pereira et al., 2012). Also, the pre-processing steps for this technique enable a better inter-subject registration for matching homologous cortical regions (Fischl et al., 1999). However, it appears that the GMV is more sensitive in examining psychopathy and violence with the present findings. Future studies should further attest to this speculation.

### Limitations

Although this was the first study to investigate how psychopathy moderated the OFC/striatum in terms of their GMV and CTh regarding violence, this study suffered from a number of limitations. Firstly, the majority of the participants were males. There has been evidence showing the difference in gender may lead to alterations in the neurological functions within antisocial individuals (Raine et al., 2011). Although the genders of the test participants were controlled for in all the analyses in the present study, future studies should recruit an equal number of male and female participants. Secondly, the information of attempted homicide and history of violent assaults (not necessarily fatal) as well as convicted homicide was not recorded in the present study. Since it is possible that the “non-violent” participants attempted homicide and were never caught or charged, the non-violent group may include the “attempted but not accused” murderers. Also, the violent group may include the “innocent” murderers. Although the conviction rate of crime has remained high (∼99%) in China, future studies should address the concern regarding attempted and convicted homicide. Moreover, violence was indexed only by homicide accusation in the present study, the history of non-fatal violent assault may also be taken into consideration in future studies. Furthermore, current findings were based on cross-sectional data. However, we hypothesized this moderated relationship based on prior literature with longitudinal data (Raine and Yang, 2006; Yang and Raine, 2009). All in all, the moderated relationship found in the present study helps to set a good foundation for future studies to confirm such a relationship with longitudinal data. Last but not least, the current findings were based on a modest sample size. However, given that the results regarding CTh and GMV in the OFC did not converge, future studies with a bigger sample size should further analyze and investigate such a difference.

## Conclusion

In spite of the limitations, the present study should be credited for some of its strength. For instance, the participants were all free from current/lifetime substance abuse or dependence, which would have otherwise complicated the structural abnormalities in the brain in relation to antisocial behaviors (Glenn and Yang, 2012). Also, the authors had furthered the findings of prior literature with their study of both CTh and GMV in both cortical and subcortical brain regions. Most importantly, the violent/non-violent individuals were stratified according to their level of psychopathy, specifically their global and sub-factors of psychopathy. This could have helped us better understand how different psychopathic traits were related to violence and specific brain regions. In summary, the present findings help us better understand the relationship between abnormalities in the brain (particularly in the OFC and striatum), psychopathy and violence, which is essential to the designing and development of intervention measures in order to mitigate the occurrence of crime. For example, since psychopathy is suggested to moderate the association among the abnormalities in the lOFC/striatum, implementing preventive programs such as the Violence Reduction Program (Wong and Gordon, 2013), which target psychopathy, may potentially reduce the incidence of violent crimes in the society.

## Author Contributions

All authors designed the study. BL analyzed the data and wrote the first draft of the manuscript. TL and YY supervised the research project and critically revised the manuscript. All authors contributed to and have approved the final manuscript.

## Conflict of Interest Statement

The authors declare that the research was conducted in the absence of any commercial or financial relationships that could be construed as a potential conflict of interest.

## Footnotes

1 http://surfer.nmr.mgh.harvard.edu

## Abbreviations

CTh: cortical thickness
GMV: gray matter volumes
lOFC: lateral orbitofrontal cortex
mOFC: medial orbitofrontal cortex
OFC: orbitofrontal cortex
PCL-R: Psychopathy Checklist-Revised
ROI: region of interest
SES: socio-economic status.
